# Understanding the Functional Needs of Patients With Cardiovascular Disease Regarding Nursing Robots Using a Kano Survey Scale: Cross-Sectional Study

**DOI:** 10.2196/92610

**Published:** 2026-08-25

**Authors:** XiuLi Wu, Aimei Kang

**Affiliations:** 1 Department of Nursing, Shandong Provincial Hospital Affiliated to Shandong First Medical University, No.324 Jingwu Road, Huaiyin District, Jinan, Shandong, 25002, China, 86 17371981751; 2 Department of Nursing, Wuhan Asia General Hospital Affiliated to Wuhan University of Science and Technology, WuHan, Hubei, China

**Keywords:** artificial intelligence, AI, Kano model, demand analysis, robot, health services research

## Abstract

**Background:**

With population aging and the growing burden of chronic diseases, the number of patients with cardiovascular disease (CVD) in China continues to rise. Nursing robots have been recognized as one potential approach to help alleviate nursing workload. However, patient-centered functional needs with respect to such robots among patients with CVD remain insufficiently explored in China.

**Objective:**

On the basis of the Kano model, this study aimed to analyze attitudes toward and demand priorities regarding nursing robot functions among patients with CVD. The findings may provide evidence to support function optimization and wider clinical application of nursing robots in China.

**Methods:**

A cross-sectional study was conducted at Wuhan Asia Heart Hospital from July 2024 to June 2025. Convenience sampling was used to enroll 156 patients with CVD. Data were collected using a self-designed questionnaire, including demographic characteristics and a 15-item Kano scale for nursing robot functions. Data were analyzed using descriptive statistics, Kano classification, better-worse coefficients, and a 4-quadrant diagram.

**Results:**

Among 15 functional items, accompanying care, consulting education, information management, and monitoring and warning were classified as must-be attributes; activity management, rehabilitation physiotherapy, drug management, security management, and logistics transfer were one-dimensional attributes; infection management, first aid skills, and nutritional care were attractive attributes; and sampling operation, personal hygiene management, and excretory care were indifferent attributes.

**Conclusions:**

Our study reveals that patients with CVD generally exhibited high acceptance of nursing robots, but there were significant differences in functional requirements. When introducing nursing robots into clinical settings, priority should be given to essential attributes such as monitoring and warning, as well as educational consultation, along with expected attributes such as medication management. At the same time, attractive attributes such as infection management can be developed as value-added features. For nondifferentiated attributes such as personal hygiene management, selective configurations can be implemented based on clinical needs to enhance the clinical applicability and patient acceptance of nursing robots.

## Introduction

Worldwide, cardiovascular disease (CVD) has emerged as one of the primary risk factors adversely affecting public health [1]. According to a global statistical report by the American Heart Association, by 2021, there were over 19.91 million deaths attributed to CVD worldwide [2]. Over the past few decades, both the incidence and mortality rates of CVD in China have exhibited a significant upward trend. The number of patients with CVD in China alone amounts to 330 million [3]. Given the substantial population in China with risk factors for CVD, the fact that its incidence and mortality rates continue to rise poses significant challenges to the Chinese health care system [4].

Parallel to the growing CVD burden is the intensifying challenge of population aging and a shortage of professional nursing resources. Older adults account for a large proportion of patients with CVD and rely heavily on long-term, specialized care, yet the nursing workforce in China is underresourced and overloaded, especially in cardiovascular departments, where nurses undertake repetitive, high-intensity tasks such as vital sign monitoring, medication guidance, and rehabilitation support [5-7]. In this context, nursing robots have emerged as a promising solution to alleviate workload, improve care efficiency, and supplement human resources in cardiovascular settings [8-11].

As a key component of intelligent health care, nursing robots have shown strong potential in supporting routine care and improving clinical delivery [12]. Current applications span health monitoring [13], daily living assistance [14,15], social companionship [16], and improvement of cognitive function [17]. Despite these advances, most research and functional designs focus on general older adult or rehabilitation populations, with limited consideration of the unique clinical needs of patients with CVD. Patients with CVD typically experience long disease courses, frequent exacerbations, polypharmacy, cardiac function–stratified rehabilitation, and elevated infection risk, creating distinct requirements for real-time monitoring, precise medication support, personalized rehabilitation guidance, and infection control [18]. Failure to address these specific needs may limit clinical translation and sustainable adoption of nursing robots in cardiovascular settings.

In recent years, research attention has shifted from technical development of robots to user needs and acceptance, with studies investigating perspectives of nursing managers, frontline nurses, and medical students [19-21]. As direct recipients of robot-assisted care, patients’ views are critical for functional design and clinical adoption, yet their needs remain understudied.

The Kano model is a framework useful for categorizing user needs and ranking functional priorities and has been applied in some health care studies [18]. It can help classify needs into must-be, one-dimensional, attractive, and indifferent attributes. Previous studies have used the Kano model in medication management [22], pulmonary rehabilitation [23], psychological care [24], and nurses’ needs for nursing robots [25]. However, few studies have used the Kano model to quantitatively explore the functional needs of patients with CVD regarding nursing robots.

Accordingly, this cross-sectional study used the Kano model to investigate the functional needs and priorities of patients with CVD regarding nursing robots. The findings may help bridge gaps between patient needs and robot function design and support the clinical translation of intelligent nursing technologies in cardiovascular care.

## Methods

### Study Design

A cross-sectional design was used to investigate functional needs regarding nursing robots among patients with CVD at a single time point.

### Research Setting

This study was conducted at Wuhan Asia Heart Hospital, the only tertiary specialized hospital in central China dedicated to the diagnosis and treatment of CVD. The hospital operates multiple departments, including cardiovascular medicine, cardiac surgery, and cardiovascular intensive care unit, with an annual patient volume of 128,000 for CVD. The patient population encompasses various conditions, such as atherosclerotic CVD, hypertensive CVD, and valvular heart disease. The distribution of patients is balanced in terms of age, educational background, disease duration, and severity, ensuring a strong representative sample that reflects the general needs of patients with CVD in central China.

### Participants

Convenience sampling was used to select patients with CVD who visited Wuhan Asia Heart Hospital from July 2024 to June 2025 as the primary study participants.

Inclusion criteria were (1) adults aged 18 years and older regardless of gender, (2) patients with a precise diagnosis of CVD (any type), and (3) voluntary participation in the study.

Exclusion criteria were (1) failure to provide valid informed consent or incomplete questionnaires, (2) individuals with significant cognitive impairment (a Mini-Mental State Examination score of <27), (3) those with decreased vision or hearing impairments making it impossible to read text or engage in conversation, (4) participants with critical acute conditions or those with end-stage disease, and (5) individuals who participated in a similar nursing robot needs survey within the previous 3 months.

### Sample Size Calculation

The minimum required sample size for the questionnaire was calculated using the following formula:


$$N=\frac{Z^{2}\times {p(1-p)}^{2}}{d^{2}}$$

A 95% CI (*Z*=1.96) and a 7.5% margin of error were applied. The minimum required sample size was 171. Considering an expected 10% invalid rate, 190 questionnaires were distributed.

### Research Tools

The survey tool comprised an online questionnaire designed with 2 sections. The first section was a general information survey that included 9 questions related to the participants’ gender, marital status, age, educational level, type of CVD, duration of illness, previous experience with nursing robots, receipt of robot services, and methods of interaction with robots. The second part featured the Kano survey scale, which addressed functional needs regarding nursing robots. On the basis of a preliminary literature analysis, we identified and analyzed tasks in nursing that could potentially be replaced by robots, classifying them into 15 demand items [26,27] using card sorting methods (Table 1). In our developed Kano demand scale, each of the 15 included items was designed to assess participants’ attitudes and preferences regarding specific functional areas of artificial intelligence (AI) nursing robots through both positive and negative questions. For instance, one question asked the following: “How do you feel if the nursing robot has/does not have a medication management function?” Each question encompassed 5 evaluation dimensions: “like it,” “must-be,” “neutral,” “accept it,” and “dislike.” Consequently, participants’ evaluations of a single item could yield 25 possible results (5 × 5), with each result corresponding to a specific attribute category (Table 2).

**Table 1.** Classification of functional areas of application of nursing robots.

Category	Application scenario
Drug management	Intravenous dispensing Drug distribution Medication reminder
Monitoring and warning	Physiological indicator measurement and early warning Ward patrol Vital sign monitoring
Logistics transfer	Transfer of patients Transfer of goods
Consulting education	Medical information query Drug guidance Disease-related knowledge guidance Prevention of misinformation and education
Security management	Prevention of falls and other accidental injuries Disaster prevention
Nutritional care	Diet and nutrition guidance Feeding meals
Activity management	Postural transition Bed-chair transfer Auxiliary walking
Excretory care	Assistance with toileting
Personal hygiene management	Organization and changing of bed linens Showering assistance Grooming (including brushing teeth, washing face, and shaving) Dressing and undressing
Accompanying care	Interpersonal interaction Accompaniment for checkups Cognitive training
Infection management	Ward cleaning, disinfection, and sterilization Wound disinfection and dressing
Rehabilitation physiotherapy	Rehabilitation knowledge guidance Development and adjustment of the rehabilitation plan Assessment of rehabilitation effect
First aid skills	Assisted CPR^a^ Oxygen and sputum absorption
Information management	Personal electronic medical information storage and record
Sampling operation	Venous blood sampling Collecting nasal or pharyngeal swabs

a CPR: cardiopulmonary resuscitation.

**Table 2.** Kano evaluation table.

Positively worded items: how would you feel if the nursing robot had X function?	Negatively worded items: how would you feel if the nursing robot did not have X function?				
	Like it	Must be	Neutral	Accept it	Dislike
Like it	Q	A^a^	A	A	O
Must be	R	I	I	I	M
Neutral	R	I	I	I	M
Accept it	R	I	I	I	M
Dislike	R	R	R	R	Q

a Definition of categories in the Kano model. A = One-dimensional quality; M = Must-be quality; I = Indifferent quality; O = Attractive quality. One-dimensional quality means user satisfaction increases linearly with the fulfillment of requirements. Must-be quality is the basic requirement; dissatisfaction occurs if unfulfilled, while satisfaction cannot be greatly improved even if fulfilled. Attractive quality brings great satisfaction when realized and causes no dissatisfaction when absent. Indifferent quality has no influence on user experience. Reverse quality leads to lower satisfaction with higher implementation. Questionable quality represents contradictory and invalid responses.

### Reliability and Validity Test

Following the completion of the questionnaire design, we invited 5 experts—3 specializing in CVD nursing and 2 specializing in AI and communication technology—to evaluate our designed Kano demand scale. They assessed the relevance of the questions using Likert scales. The item-level content validity index for the Kano demand questionnaire ranged from 0.71 to 1.0, whereas the scale-level content validity index was 0.8. We assessed the effectiveness of the questionnaire using the Cronbach α coefficient, which was found to be 0.929. Additionally, internal consistency was evaluated through the retest coefficient, which yielded a value of 0.871. These results indicate that the effectiveness and reliability of the questionnaire were acceptable.

### Data Collection and Analysis

Participants were enrolled at Wuhan Asia Heart Hospital from July 2024 to June 2025. First, after completing the questionnaire design, the researchers created a survey using an online platform called Wenjuanxing (“Questionnaire Star”). Subsequently, the study objectives, content, risks, and benefits were explained to the participants, who voluntarily signed an electronic informed consent form. Finally, patients were guided to complete the questionnaire via mobile phones or tablets, with a completion time of 15 to 25 minutes. During this process, only the definitions of questionnaire items were explained, and no intervention was made on the participants’ choices. After the submission of the questionnaires, data collection began. During this phase, questionnaires exhibiting incomplete responses or evident logical contradictions were identified as invalid and excluded from subsequent analytical procedures.

SPSS (version 26.0; IBM Corp) was then used for data analysis. General data were described using descriptive statistics (numbers and percentage distributions). To comprehensively and accurately delineate the functional requirements regarding nursing robots among patients with CVD, 3 analytical approaches were used: the Kano model classification, the better-worse coefficient analysis for prioritization, and the construction of a Kano demand quadrant diagram.

First, cross-tabulation was performed on the positive and negative responses to each function in the questionnaire, categorized into 4 types (must attributes [M], one-dimensional attributes [O], attractive attributes [A], and indifferent attributes [I]). The frequency and proportion of each function being classified as different attributes were statistically analyzed, with the attribute type with the highest proportion serving as the final attribute classification for that function.

Second, we used the better-worse coefficient analysis method [23] to prioritize questionnaire items, thereby clarifying the impact level of specific functional domains on survey participants. This analytical method consists of “better” coefficients and “worse” coefficients, where the absolute values of the coefficients (ranging from 0 to 1) reflect satisfaction or dissatisfaction with the provision of services. Specifically, the closer the “better” value is to 1, the more satisfied patients are when the target attribute is provided. Conversely, the closer the absolute value of the “worse” coefficient is to 1, the higher the dissatisfaction level when the target attribute is not provided. The calculation formula for the coefficients is as follows:


$$\begin{array}{rl} \text{Better coefficient} & =\frac{A+O}{A+O+M+I},\\ \text{Worse coefficient} & =\frac{O+M}{A+O+M+I} \end{array}$$

Finally, a 4-quadrant diagram was constructed with the “better” coefficient as the vertical axis and the absolute value of the “worse” coefficient as the horizontal axis. Specifically, “O” attributes located in the first quadrant (upper right region) exhibit both the “better” coefficient and the absolute value of the “worse” coefficient exceeding the mean, representing desirable needs that warrant greater attention and should be prioritized. “A” attributes with high “better” coefficients and low absolute values of the “worse” coefficient are situated in the second quadrant (upper left region), indicating desirable needs. “M” attributes in the fourth quadrant (lower right region) exhibit lower “better” coefficients and higher absolute values of the “worse” coefficient, representing essential attributes. “I” attributes in the third quadrant (lower left region) exhibit both lower “better” coefficients and lower absolute values of the “worse” coefficient, indicating nondifferentiated needs. These needs are not significant to patients and may be omitted or do not require improvement [28].

### Ethical Considerations

The ethics committee of Wuhan University of Science and Technology (2024-096-02) approved this study, which adheres to the ethical standards outlined in the Declaration of Helsinki. Participation in this survey was voluntary and anonymous, and the participants provided informed consent. Participants could refuse or withdraw at any time, and the information collected was used solely for academic research purposes and not for commercial gain. All data were exclusively collected for this study, encrypted using a dual-password system (with passwords kept separately by 2 researchers), and stored on a hospital-specific research data server. The server undergoes regular security audits and backups. Data leakage, tampering, or use for other purposes are strictly prohibited. Upon completion of the survey, the data were archived in the hospital’s research data platform for a period of 5 years, after which they will be uniformly destroyed in accordance with regulations.

## Results

### Basic Information of Survey Participants and Their Understanding of Nursing Robots

From July 2024 to June 2025, this study distributed 190 questionnaires, of which 6 (3.2%) were not completed due to privacy concerns. Of these 190 questionnaires, after excluding 28 (14.7%) that were invalid, a total of 156 (82.1%) valid questionnaires were collected. Although the final sample was slightly below the calculated target, post hoc evaluation indicated that the sample size was adequate for Kano classification and coefficient analysis, which rely on proportion comparisons rather than mean estimation.

The survey primarily focused on the Wuhan area of Hubei Province. The demographic characteristics of the study participants are summarized in Table 3. A total of 75.6% (118/156) of the participants were male, 45.5% (71/156) were aged over 60 years, 52.6% (82/156) had a bachelor’s degree or higher, and 59% (92/156) had a disease duration of less than 10 years. Among the participants, 48.1% (75/156) had experience using nursing robots, 68.6% (107/156) had received nursing robot services, and 45.5% (71/156) preferred interactive methods such as images and videos.

### Demand Attribute Classification Analysis

Through the Kano model, we analyzed the requirements regarding nursing robots for patients with CVD across 15 functional areas. As shown in Table 4, based on the final survey results, we identified 3 items as “M” attributes: monitoring and warning, consulting education, and information management. Drug management and rehabilitation physiotherapy were categorized as “O” attributes. “A” attributes included 5 items: security management, logistics transfer, nutritional care, infection management, and first aid skills. The presence of these functional areas would make the nursing robots more acceptable to patients. The remaining 5 items were categorized as “I” attributes, which are not important to patients. These were excretory care, personal hygiene management, accompanying care, activity management, and sampling operation.

**Table 3.** Basic participant information (N=156).

Variable	Participants, n (%）
Sex	
Male	118 (75.6)
Female	38 (24.4)
Marital status	
Married	111 (71.2)
Unmarried	45 (28.8)
Age (y)	
18-25	37 (23.7)
26-30	4 (2.6)
31-40	36 (23.1)
41-50	15 (9.6)
51-60	3 (1.9)
>60	71 (45.5)
Educational background	
Primary school and lower	19 (12.2)
Junior middle school	31 (19.9)
High school or technical secondary school diploma	24 (15.4)
Undergraduate or junior college	73 (46.8)
Master’s degree or higher	9 (5.8)
Cardiovascular disease type	
Atherosclerotic cardiovascular disease	44 (28.2)
Hypertensive cardiovascular disease	19 (12.2)
Valvular heart disease	29 (18.6)
Arrhythmia	46 (29.5)
Cardiomyopathy	11 (7.1)
Pericardial disease	5 (3.2)
Other	2 (1.3)
Duration of disease (y)	
<10	92 (59)
10-20	36 (23.1)
>20	28 (17.9)
Previous use of nursing robots	
Yes	75 (48.1)
No	81 (51.9)
Receipt of robot services	
Yes	107 (68.6)
No	49 (31.4)
Methods of interaction with robots	
Text input	47 (30.1)
Images and videos	71 (45.5)
Speech recognition	29 (18.6)
Other	9 (5.8)

**Table 4.** Classification of structural attributes of functional requirements for nursing robots among patients with cardiovascular disease (N=156)^a^.

Item	Attribute classification						Final attribute classification
	M^b^, n (%)	O, n (%)	A, n (%)	I, n (%)	R, n (%)	Q, n (%)	
Drug management	2.56	35.9	26.28	17.95	16.03	1.28	O
Monitoring and warning	39.1	10.26	16.03	25	8.33	1.28	M
Logistics transfer	24.36	17.31	28.21	19.87	8.33	1.92	A
Consulting education	28.21	12.18	26.28	20.51	11.54	1.28	M
Security management	13.46	28.21	30.13	19.87	7.69	0.64	A
Nutritional care	5.77	12.82	50.64	21.15	8.33	1.28	A
Activity management	5.41	26.35	24.32	37.16	6.08	0.68	I
Excretory care	3.85	5.13	15.38	66.03	7.69	1.92	I
Personal hygiene management	3.85	7.05	14.1	67.31	7.05	0.64	I
Accompanying care	26.28	13.46	14.74	34.62	9.63	1.28	I
Infection management	4.49	6.41	53.85	27.56	7.05	0.64	A
Rehabilitation physiotherapy	3.21	35.9	19.87	30.77	8.97	1.28	O
First aid skills	5.13	11.54	47.44	24.36	8.97	2.56	A
Information management	37.82	9.62	24.36	17.95	8.97	1.28	M
Sampling operation	7.69	7.69	22.44	52.56	8.97	0.64	I

a Percentages are presented without subgroup n within cells. Each cell reflects joint results from two-dimensional Kano evaluation, and separate subgroup counts may cause misinterpretation. The overall sample size is reported in the main text.

b Definition of categories in the Kano model. A = One-dimensional quality; M = Must-be quality; I = Indifferent quality; O = Attractive quality; One-dimensional quality means user satisfaction increases linearly with the fulfillment of requirements. Must-be quality is the basic requirement; dissatisfaction occurs if unfulfilled, while satisfaction cannot be greatly improved even if fulfilled. Attractive quality brings great satisfaction when realized and causes no dissatisfaction when absent. Indifferent quality has no influence on user experience. ontradictory and invalid responses.

### Better-Worse Coefficient Analysis

**Figure 1.**
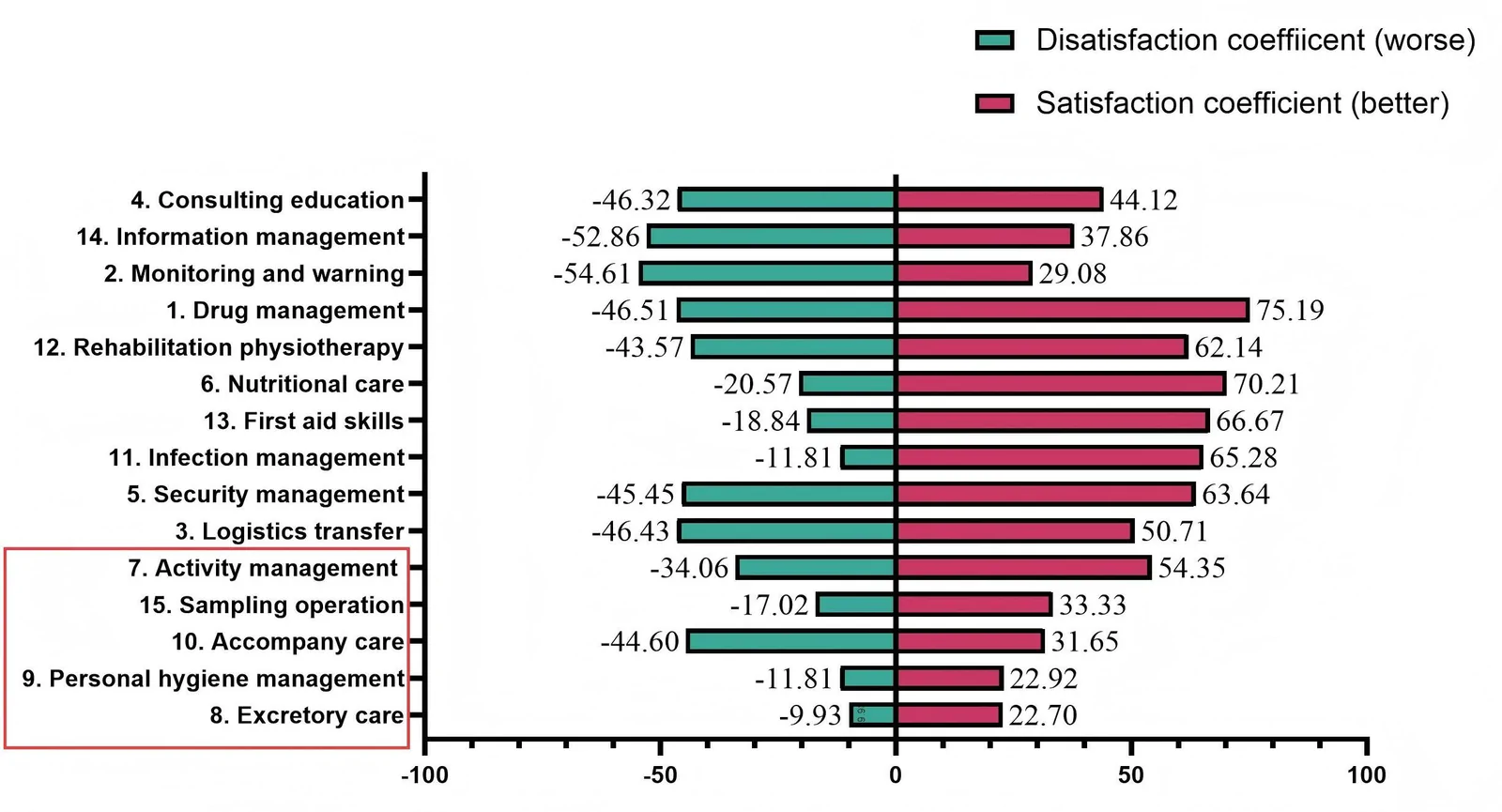
Satisfaction index and dissatisfaction index of functional requirements regarding nursing robots of patients with cardiovascular disease.

To better visualize the functional differences across domains, we also created a comparative bar chart of “better” and “worse” coefficients for the functional requirements of patients with CVD regarding nursing robots. In Figure 1, among the top 10 functional areas, drug management, rehabilitation physiotherapy, nutritional care, first aid skills, infection management, security management, and logistics transfer had relatively high satisfaction index values ranging from 0.5 to 0.8. This indicates that, when the nursing robot possesses these functions, it will achieve high levels of satisfaction and acceptance. The other 3 functions (consulting education, information management, and monitoring and warning), although they had lower satisfaction index values, exhibited a high absolute dissatisfaction index value. This suggests that, if these requirements are not met, users will be highly dissatisfied regardless of how well other functions are performed, leading to a lack of increased favorability toward the product.

### Kano Demand Quadrant Diagram

**Figure 2.**
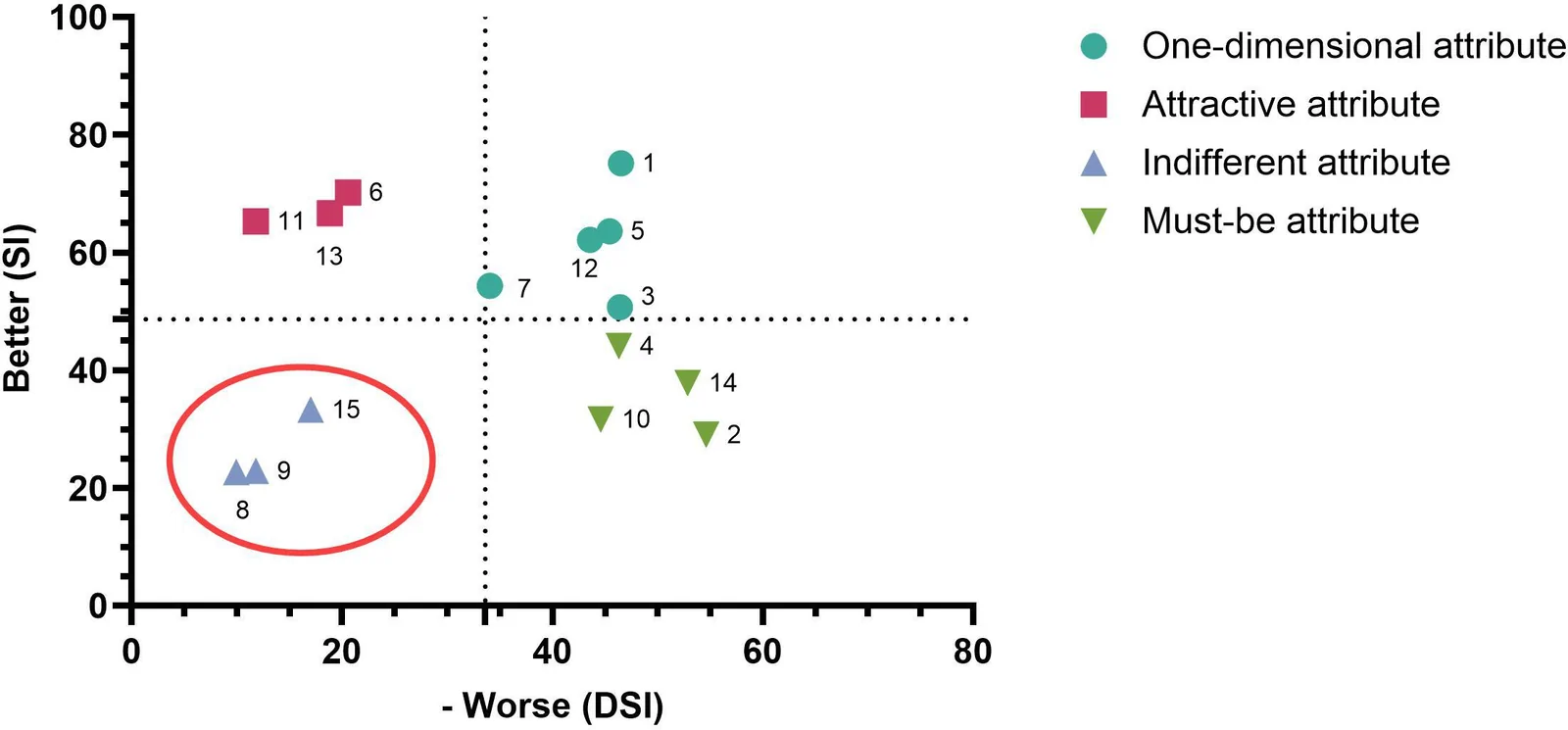
Kano quadrant diagram of the demands of patients with cardiovascular disease regarding nursing robot functions. DSI: dissatisfaction index; SI: satisfaction index.

We plotted a Kano demand quadrant diagram (Figure 2) using the better-worse coefficients. The results indicated that 4 items were categorized within the must-be attribute quadrant: monitoring and warning, consulting education, accompanying care, and information management. Five items were classified in the one-dimensional attribute quadrant: drug management, security management, logistics transfer, activity management, and rehabilitation physiotherapy. The attractive attribute quadrant included 3 items: nutritional care, infection management, and first aid skills. The remaining 3 items were placed in the indifferent attribute quadrant: excretory care, personal hygiene management, and sampling operation. Compared to the straightforward classification of needs presented in Figure 1, Figure 2 offers a more nuanced prioritization of each functional area, resulting in more precise outcomes. The findings illustrated in Figure 2 further corroborate that the functional areas of sampling operation, excretory care, and personal hygiene management are of relatively low priority.

## Discussion

### Acceptance and Interaction Preference Characteristics Regarding Nursing Robots Among Patients With CVD

In this study, 68.6% (107/156) of participants with CVD had received nursing robot–assisted care, and 48.1% (75/156) reported prior exposure to the technology. This high familiarity likely stems from the 2-year clinical deployment of the Noah nursing robot in the study hospital’s cardiology ward, which allowed patients to gradually build trust in this care model. This preexposure may have positively biased participants’ attitudes toward nursing robots and, in turn, their perceptions of functional needs and Kano classification results. Our findings should therefore be generalized with caution to settings without routine nursing robot use and further validated in larger, multicenter cohorts across different hospital tiers and regions.

For interaction preferences, 45.5% (71/156) of patients preferred visual or video-based interaction, 30.1% (47/156) favored text input, and only 18.6% (29/156) chose voice recognition. This pattern is closely tied to the 45.5% (71/156) of older adult patients in our cohort: visual and video formats deliver medication guidance and rehabilitation instructions more intuitively for older adults, whereas many patients also voiced concerns about the accuracy of voice recognition in busy wards and associated privacy risks, leading to a preference for traceable, visual interaction.

Consistent with previous work [29], voice interaction still has clear potential for efficient human-machine communication. Our findings suggest that nursing robot design should prioritize multimodal interaction, with targeted optimization of voice recognition for older adult patients’ dialects plus accessible interaction record functions to strengthen privacy and security for users.

### Structural Analysis of the Functional Needs of Patients With CVD Regarding Nursing Robots Based on the Kano Model

Using the Kano model, we categorized 15 core nursing robot functions into 4 attributes: must be (M), one-dimensional (O), attractive (A), and indifferent (I). This classification clarifies the clinical positioning and optimization priorities of each function, providing evidence-based guidance for the design and clinical deployment of nursing robots for CVD care.

“M” attributes included monitoring and early warning, information management, and consultation and education, all of which align with the core needs of long-term monitoring and self-management for patients with CVD. Notably, the monitoring and early warning function had an absolute “worse” coefficient of 54.6, meaning that more than half of the patients would report reduced satisfaction if this function were absent. This is consistent with the chronic, fluctuating nature of CVD, where real-time monitoring and timely warning are critical to prevent adverse events [30]. These core basic functions should be prioritized in robot design as they form the foundation of clinical acceptance and align with the shift toward proactive, precision smart nursing.

Medication management and rehabilitation physiotherapy were classified as “O” attributes, with “better” coefficients over 60 for both, showing that optimizing these functions directly improves patient satisfaction. Patients with CVD typically take an average of 3.2 concurrent medications, with complex regimens carrying a high risk of dosing errors and nonadherence [31], explaining the strong demand for integrated medication reminders, dose verification, and supply alerts. For rehabilitation, patients preferred personalized plans stratified by New York Heart Association cardiac function class over standardized programs, reflecting the need for personalized, rather than one-size-fits-all, smart nursing support.

Infection management, nutritional care, and first aid skills were identified as “A” attributes. While not mandatory for patients, these functions drive significant improvements in satisfaction. The infection management function had a “better” coefficient of 65.3, which aligns with the higher infection risk in immunocompromised patients with CVD [32]. Personalized nutrition plans tailored to lipid and glucose levels also fit the model of precision nutritional support, making these functions useful value-added features for high-acuity settings such as cardiovascular intensive care units.

Personal hygiene management, excretion care, and sampling operations were classified as “I” attributes, with minimal patient demand for robot delivery of these services. This low demand is likely driven by 2 key factors: privacy concerns regarding highly personal care tasks and patient preference for functions that directly improve self-care safely rather than high-risk, specialized operations. This preference for robot involvement in noninvasive, nontechnical care aligns with findings of previous studies [17,33,34]. Routine large-scale deployment of invasive functions such as blood sampling is therefore not recommended; these may only be selectively used in specific settings such as isolation wards. Optimizing privacy protection features will also be key to addressing patient trust concerns around these functions.

Overall, the clinical deployment of nursing robots for CVD care should be guided by patient needs following a hierarchical optimization framework: prioritizing essential must-be functions, optimizing one-dimensional functions to improve care quality, adding attractive functions as value-added features, and limiting indifferent functions to scenario-specific use. This framework aligns robot design and technical development with clinical needs, supporting the practical advancement of smart nursing.

### Implications for Clinical Practice and Future Research

Our findings have direct implications for the clinical deployment of nursing robots in Wuhan and beyond, as well as for the advancement of patient-centered intelligent cardiovascular nursing. Locally, the hierarchical priority structure we identified can be used to refine existing nursing robot programs in Wuhan hospitals, with prioritized ward deployment of high-demand functions such as monitoring and early warning, medication management, and nutritional support to align services with patient needs.

For routine nursing practice, integrating robots into high-demand processes such as medication monitoring can standardize care, reduce human error, and improve continuity of care. Rather than large-scale changes to existing nursing workflows, we recommend a phased approach: piloting high-priority functions, evaluating safety and acceptability, and then gradually expanding successful modules.

These findings also highlight key directions for future research. Larger multicenter studies are needed to validate the generalizability of these demand patterns, whereas subgroup analyses can clarify heterogeneous needs across patient groups. Longitudinal studies evaluating the safety, usability, and clinical outcomes of robot-assisted care will also be critical to support evidence-based translation into practice.

Ultimately, this study addresses a key gap between nursing robot technical development and real-world clinical needs by centering patient perspectives in function design. The proposed hierarchical framework supports a shift from generalized robot deployment to precision, patient-centered intelligent care, which has the potential to reduce nursing workload, improve care standardization, and enhance patient safety and satisfaction in cardiovascular care.

### Limitations

This study has several limitations. First, the sample was limited to 1 hospital in Wuhan using convenience sampling, which may reduce generalizability. Second, subgroup analyses by age, educational level, or disease severity were not performed, although age stratification was explored and showed limited between-group differences in this cohort. Third, this was a cross-sectional self-reported survey, which may be subject to response bias. Future studies should use larger, multicenter samples; perform stratified analyses; and include longitudinal follow-up to strengthen the evidence base.

### Summary

The presence of robots and the advent of more advanced robotics technologies have led to an increase in studies assessing user acceptance of robots and needs analyses in the past 2 years. Wichmann et al [35] showed that perceived usefulness, attitude, and trust significantly and positively predict willingness to use AI and use behaviors in short- and long-term treatment. In our view, clarifying the needs of patients can effectively improve users’ acceptance of AI and may become a shortcut to achieving intelligent medical care in China. Our study explores the differences in prioritizing needs regarding nursing robots from the perspective of patients with CVD. This paper suggests that researchers should emphasize understanding the patient experience, which is crucial for advancing various fields, including counseling, education, companion care, and more. We hope that our research can help nursing robot developers design targeted functional services for patients with CVD, promoting the continuous development and application of nursing robotics as well as innovation and progress in the medical field.

## Abbreviations

AI: artificial intelligence
CVD: cardiovascular disease
